# Categorization of Jammers for Spaceborne SAR Systems

**DOI:** 10.3390/s26061757

**Published:** 2026-03-10

**Authors:** Martin Chiari, Thomas Jagdhuber, Rainer Speck, Madhu Chandra

**Affiliations:** 1 Microwaves and Radar Institute, German Aerospace Center, 82234 Weßling, Germany; 2 Faculty of Applied Computer Science, University of Augsburg, 86159 Augsburg, Germany; 3 Faculty of Electrical Engineering and Information Technology, University of Technology Chemnitz, 09111 Chemnitz, Germany

**Keywords:** radar, SAR, jamming

## Abstract

**Highlights:**

**What are the main findings?**

- Jammers can be categorized based on the physical properties of the jamming signal.
- Three basic categories are jamming by transmission power, jamming by signal shape, and intelligent jamming.

**What are the implications of the main findings?**

- Jamming effects cannot always be assigned to specific categories solely by their appearance in the resulting image.
- Based on jamming categories, countermeasures can be developed.

**Abstract:**

SAR systems are widely used in military, scientific, and commercial missions. The use of these systems makes jamming a focal point for users. To reduce the impact of jamming, this phenomenon needs to be better understood. This paper suggests a method for the categorization of jamming mechanisms in synthetic aperture radar (SAR) systems that builds on the physics of SAR acquisitions and jamming signals. It investigates the effects of different types of jammers on SAR images and systematically categorizes them. The categorization is based on the mathematical representation of the physics of the jamming signal and the SAR image formation process. For all suggested categories, jammers are simulated based on a simple, generic scene to investigate their effects on the resulting SAR image. These effects can vary across a wide range—from adding background noise to unfocused responses similar to point target scattering. Moreover, an intelligent jammer is even able to generate false targets virtually or to reduce the signal intensity of real targets by retransmitting signals fully adapted to the image acquisition scenario. Categorizing jammers is a bottom-up task best based on physics. It will be essential in the future when the sky is crowded with constellations of satellites and interference levels increase significantly and become commonplace.

## 1. Introduction

In recent decades, the number of spaceborne SAR systems has increased significantly worldwide. In the scientific sector, the European Union launched the Sentinel 1 Mission [1] in 2014, Germany has been operating TanDEM-X [2] since 2010, and Canada has been using the Radarsat constellation [3] for the last 5 years. Also, an increasing number of commercial SAR systems is noticeable [4]. In addition to these civilian systems, a large number of systems for military use are currently operating or under development.

Due to the increasing total number of image acquisitions by spaceborne systems and constellations, a problem has become increasingly prominent to both image acquisition specialists and image interpretation specialists. A greater number of images are being influenced by jamming signals, which can reduce image quality significantly. This problem can be so severe that images become unusable.

These jammers are assumed to be unintended in most cases and are mainly caused by the increasing use of microwave transmitters and wireless communication technologies [5]. However, in military contexts, intended and planned jamming is a possibility in modern warfare [6]. Moreover, the required technologies have become less expensive and readily available, which also fosters jamming activities.

**Figure 1:**
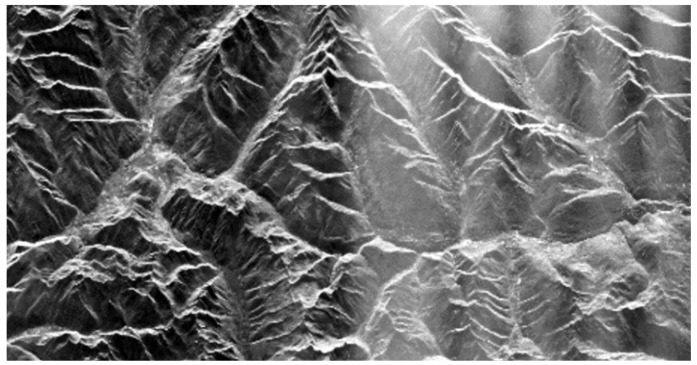
SAR image taken by the TerraSAR-X satellite with jamming effects seen on the right-hand side of the image [7].

Figure 1 shows an example of an image with a significant level of noise on the right-hand side caused by a jammer. The jamming was unintended and caused by a misconfigured ground-based radar system.

The first step to address the challenges of investigating jamming is to obtain a better understanding of the methods and mechanisms of jamming during the SAR image acquisition process. Until now, the problem has been addressed only sporadically and is scattered among a small number of publications, such as [6,7,8,9,10]. These publications focus only on individual aspects of a topic that is indeed multifaceted.

This study provides a broader view and suggests an alternative categorization scheme for jammers, as in [11]. The proposed categorization scheme is not based on jamming effects in the image but on the mathematical description of the jamming signal. To achieve this goal, the categorization is defined by two signal parameters: signal power and signal shape. Based on this categorization, the study investigates a series of matching jamming mechanisms systematically through a multi-modal forward simulation experiment to model pure jamming effects. It does not analyze and remodel jamming effects in acquired SAR images using a reverse, backward approach.

The three basic categories are defined and presented in Section 2. Based on this categorization, jammers are described, formulated and simulated for each jamming category in Section 3. The results are discussed in Section 4. The article ends with conclusions in Section 5.

## 2. Categorization of Jammers

Intended or unintended, a jammer transmits a signal in the direction of the sensor. The signal received by the sensor can be defined, in general, as

(1) $$s_{jam,Rx}=P_{jam,Rx}\cdot s(f_{0},\tau ,T,B,t).$$

Equation (1) consists of two terms. The first term, $P_{jam,Rx}$, is the power of the received signal. The second term characterizes the signal shape, which is described by the analog parameters of carrier frequency *f*_0_, pulse duration *τ*, pulse repetition interval *T*, and bandwidth *B* over time *t*. Different effects in the final SAR image can be attributed to each term.

**Figure 2:**
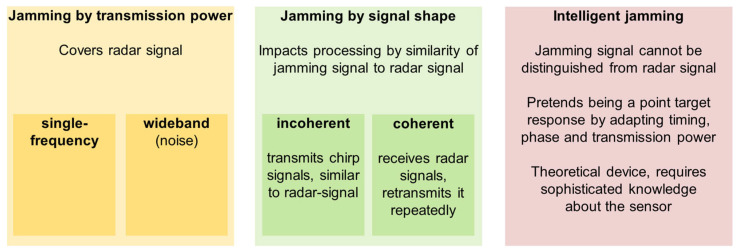
Categorization scheme for jammers, defining three fundamental categories based on the physical properties of the jamming signal.

Based on these terms, a categorization scheme is suggested, as shown in Figure 2. Three basic categories are defined. The first category is jamming by transmission power, representing the first part of Equation (1). This category contains all types of jammers that try to cover the radar signal by transmitting jamming signals with high transmission power. In this category, two subcategories can be established. Based on the type of applied signal, the subcategories are single-frequency jammers and wideband jammers.

Jammers that use a modulated signal to influence signal processing at the sensor level belong to the second category: jamming by signal shape. This represents the second part of Equation (1). Within this category, two subcategories can again be defined. If the jamming signal is generated independently of the radar signal, the transmitter is an incoherent jammer. In the case that the device receives and retransmits the radar signal, it is a coherent jammer.

The jammer that retransmits the radar signal and adapts the signal’s timing, phase, and power represents the third category: an intelligent jammer. This category contains jammers that are able to generate additional or false information and represents the perfect combination of both parts of Equation (1).

## 3. Simulation of Jammers

In this section, the jammers categorized in the previous section are described and simulated in order to characterize their effects on the resulting SAR images. These simulations are based on prior work presented in [12].

Most SAR systems use a chirp signal in baseband that is defined by

(2) sch(t)=Reejπ(κt+2fstart)⋅t=cosπ(κt+2fstart)⋅t

with the chirp rate *κ*, which is defined by the ratio of bandwidth *B* to pulse duration *τ*, and a starting frequency *f_start_*.

After being reflected by a point target and travelling back at the speed of light *c*_0_ to the sensor [13], the receiver records a complex signal in baseband as

(3) $$s_{Rx}(t)=\sqrt{P_{Rx}}\cdot \frac{1}{4}\cdot e^{j\left[\pi (\kappa \left(t-\frac{2R}{c_{0}}\right)+2f_{start})\left(t-\frac{2R}{c_{0}}\right)\right]}\cdot e^{j\left[-2\pi f_{0}\frac{2R}{c_{0}}\right]}$$

where the received signal power *P_Rx_*, derived from the radar equation in [14], depends on the transmission power of the sensor *P_Tx_*, the radar cross section of the target *σ*, and the distance from the target to the sensor *R*.

**Figure 3:**
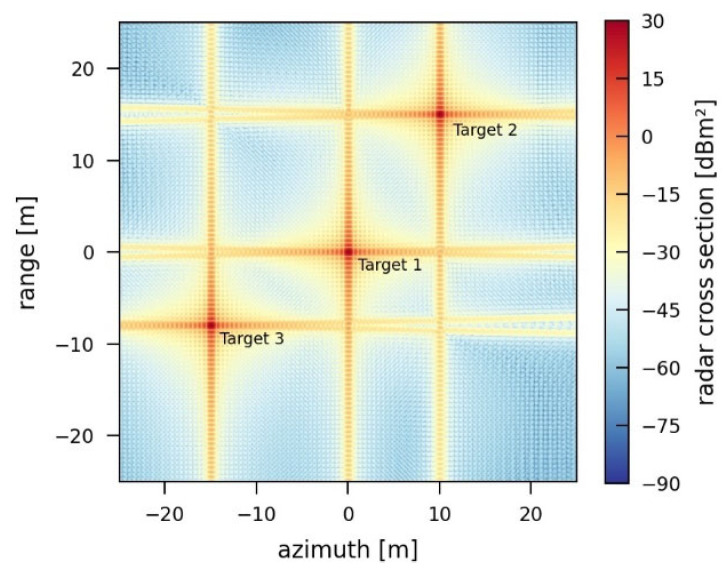
Simulated SAR image of the defined reference scenario without jamming containing three-point targets, each with a radar cross section of 1000 m^2^.

As a basis for further simulations, a reduced, synthetic reference scenario is defined. Due to the focus being on the effects caused by the jammers, a complex scene is not required. The reduced scene is required to focus directly and solely on the jamming effects, which are not influenced by other parameters of a real sensor. Table 1 lists the corresponding parameters. In the scenario, three-point targets (Targets 1–3) with a 1000 m^2^ radar cross section are placed. The jammer is always located at the coordinates (0 m, −25 m) and lies in the middle of the azimuth axis and 25 m below the image. The resulting SAR image, as a reference without any jamming, is shown in Figure 3.

In this work, the quality of an image is measured using the following image quality parameters: spatial resolution, peak sidelobe ratio (PSLR), integrated sidelobe ratio (ISLR), and the background noise level. The parameters are measured for each target.

For the reference simulation scenario, the corresponding values are shown in Table 2. In the simulated reference image, background noise is not considered since this is an ideal case. The simulated image is calibrated in such a way that the resulting radar cross section of Target 1 equals the given RCS of 1000 m^2^/30 dBm^2^. All parameters and resulting target properties in all following simulations are specific to the simulated, generic scenario. A real-world scenario is much more complex and contains a lot more parameters, but the generic simulations should provide a qualitative approach to the expected effects and allow for the comparison of the simulation results.

### 3.1. Jamming by Transmission Power

#### 3.1.1. Single-Frequency Jammer

The simplest jammer consists of a transmitter that transmits a monofrequent, continuous wave with a defined transmission power. Depending on this transmission power, the jamming signal is able to dominate the signal received by the sensor. This type of jammer only impacts one frequency of the radar signal. During processing, however, the signal energy is spread over the whole image due to the correlation between the received signal and the radar signal. The resulting jamming noise is extracted by subtracting the reference image (Figure 3) from the image under investigation. As a parameter for characterizing the strength of the jamming effect, the jamming-induced noise level (JINL) is introduced. It is measured over the entire image by calculating the median of the resulting noise image. The JINL can be used to characterize jamming effects that are spread over the entire image in reduced scenarios, as no additional effects—such as those caused by nonlinearities, additional noise, or receiver clipping—can have unwanted effects, which is the main scope of this study. It can serve as an additional metric for a deeper understanding of jamming effects. Its usability depends on the goal of the specific investigation.

**Figure 4:**
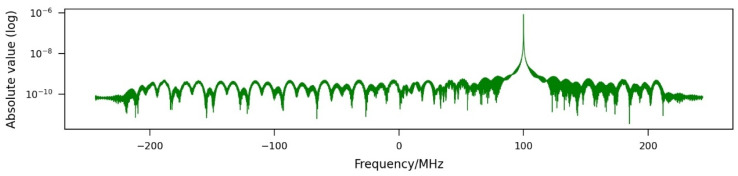
Spectrum of a jammed range profile in the middle of the synthetic aperture with a single-frequency jamming signal at 9.7 GHz (100 MHz in baseband) and a transmission power of 5 W.

**Figure 5:**
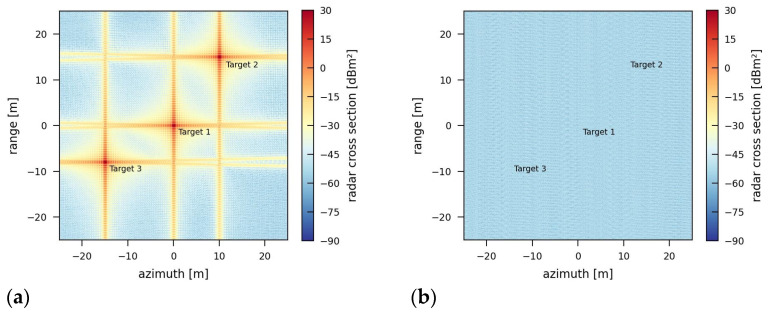
Target areas in a SAR image jammed by a 5 W single-frequency jammer in (**a**) the jammed image and (**b**) the image of the jamming signal only.

For the first simulation, the jammer constantly transmits a signal at 9.7 GHz with a transmission power of 5 W. It is received by the sensor together with the radar signal. In the spectrum of the received signal, shown in Figure 4, the jamming signal is clearly visible. Figure 5 shows the result for the first simulation. The jamming effect is not very strong; the JINL is about −52.4 dB.

**Figure 6:**
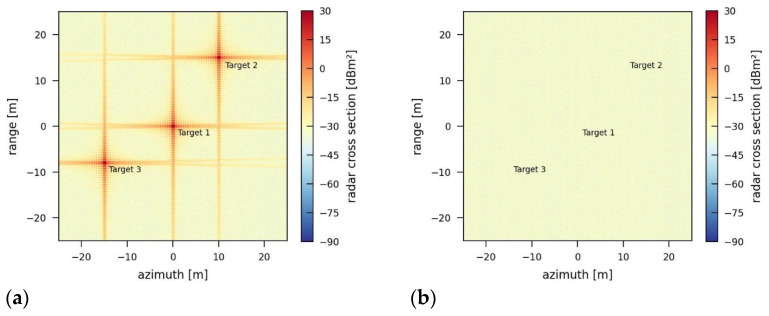
Target areas in a SAR image jammed by a 500 W single-frequency jammer in (**a**) the jammed image and (**b**) the image of the jamming signal only.

The second simulation uses the same jammer characteristics but with a transmission power of 500 W. In Figure 6, the jammed image shows an increased noise level due to the added energy of the jamming signal. This leads to a jamming-induced noise level (JINL) of about −32.4 dB. The other image quality parameters are not affected by this kind of jamming signal. Compared to the JINL in Figure 5 under a 5 W jammer, the JINL caused by the 500 W jammer also increased by 20 dB.

#### 3.1.2. Wideband Jammer

This type of jammer transmits a noise signal with a defined bandwidth and transmission power. Compared to the single-frequency jammer, a larger part of the radar signal is affected by the jammer, and the wideband signal spreads more energy over the resulting image. Therefore, the influence on the signal-to-noise ratio is stronger. The first simulation uses a jamming signal of 5 W and a bandwidth of 150 MHz.

**Figure 7:**
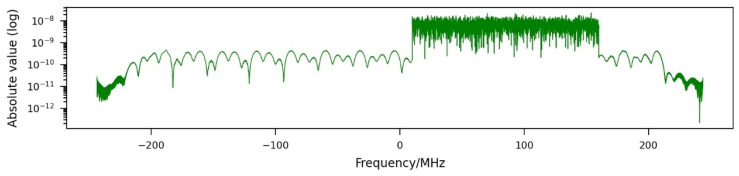
Spectrum of a range profile jammed by a wideband jammer using a transmission power of 5 W and a bandwidth of 150 MHz.

The spectrum of a range profile shown in Figure 7 clearly reveals the wideband jamming effect. Compared with Figure 4, a wider range of frequencies is affected. This leads to a higher amount of energy contained in the signal.

**Figure 8:**
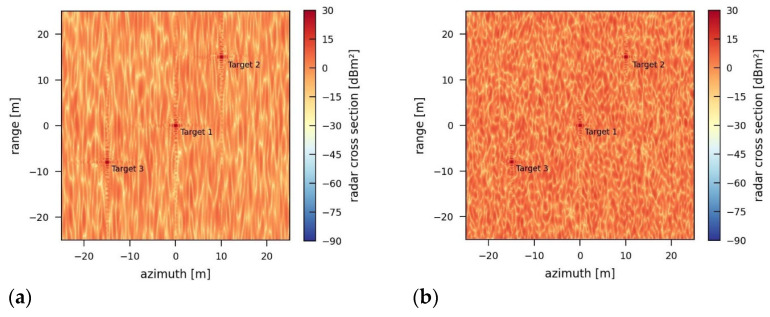
Simulation of the reference scene influenced by a wideband jammer using a transmission power of 5000 W and a bandwidth of (**a**) 50 MHz and (**b**) 150 MHz.

The amount of energy depends not only on the transmission power but also on the bandwidth of the jamming signal. Figure 8 presents SAR images for a jamming signal with a power of 5000 W using different bandwidths of 50 MHz and 150 MHz. The jamming-induced noise level increases with the bandwidth from −2.02 dB (50 MHz) to 2.72 dB (150 MHz). More power and higher bandwidth will increase the noise level in such a way that the point targets in the scene might be fully covered by the noise in the end.

### 3.2. Jamming by Signal Shape

Focusing on the second term in Equation (1), another kind of jamming mechanism is possible. This type of jammer impacts the data processing itself using signals similar to the radar signal. Two types of jamming achieved by signal shape are described here.

#### 3.2.1. Incoherent Jammer

**Figure 9:**
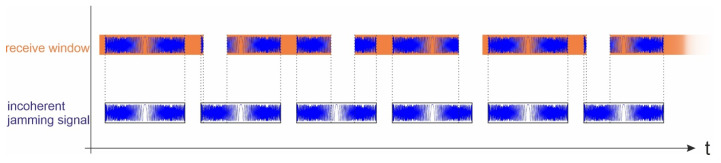
Timing of the incoherent jammer transmitting a chirp signal during the SAR acquisition at the sensor.

The incoherent jammer transmits, independently and repeatedly, a signal that is similar in shape to the radar signal. In most spaceborne SAR systems, a chirp signal is used [15]. The jammer also transmits a frequency-modulated chirp signal but does not know the exact signal properties (bandwidth, pulse duration, or pulse repetition frequency) used by the sensor. A timing scheme for the incoherent jamming during SAR acquisition is shown in Figure 9.

The position in time of the jamming signal in the sensor’s receive window depends on the properties and parameters of the sensor and the jammer.

**Figure 10:**
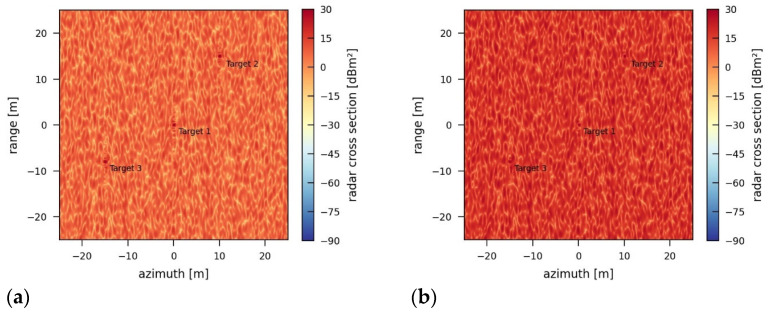
SAR images jammed by an incoherent jammer using a bandwidth of 150 MHz, a repetition frequency of 20 kHz, a pulse duration of 5 µs, and transmission power of (**a**) 50 W and (**b**) 500 W.

**Figure 11:**
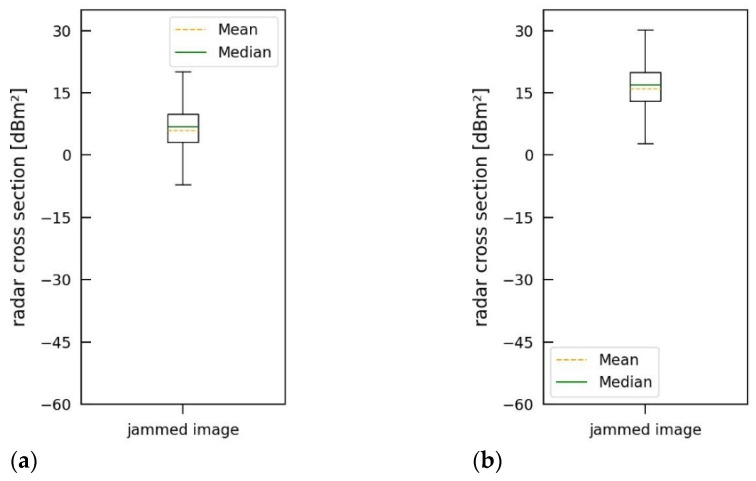
Boxplots for SAR images jammed by an incoherent jammer using a bandwidth of 150 MHz, a repetition frequency of 20 kHz, a pulse duration of 5 µs, and transmission power of (**a**) 50 W and (**b**) 500 W.

Simulation results for an incoherent jammer using different transmission powers are provided in Figure 10. The median jamming-induced noise level is 7.18 dB for 50 W and 17.96 dB for 500 W of transmission power. These values can also be seen in the corresponding boxplots in Figure 11, which are derived from the jammed images. From these plots, further statistical properties of the jamming can be determined.

Although the bandwidth of the jamming signal is much lower than the bandwidth of the radar signal, the impact of the jammer on the SAR image is distinct and strong. Compared with Figure 8b (JINL: 2.72 dB), significantly less transmission power is required to increase the noise level such that the point targets are hardly recognizable and the resulting jammed image is no longer meaningful.

**Figure 12:**
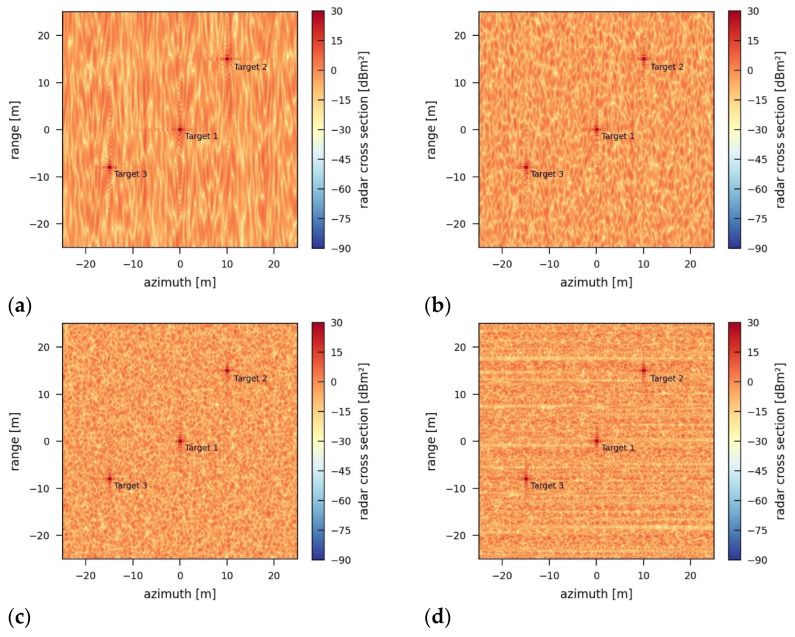
SAR images jammed by an incoherent jammer using different bandwidths of (**a**) 50 MHz, (**b**) 150 MHz, (**c**) 300 MHz, and (**d**) 424 MHz (original sensor bandwidth) and a transmission power of 5 W.

Varying the bandwidth of the jamming signal changes the characteristic spatial patterns of the jamming effects in the jammed image. Figure 12 depicts the jammed images for bandwidths that approach the bandwidth of the originally transmitted radar signal. The more the jammer bandwidth fits the radar system’s bandwidth of 424 MHz, the less vertical and the more horizontal the spatial patterns and structure of the background noise in the image become.

The reason for this structural outcome and behavior is the mismatch in signal correlation between the recorded signal and the transmitted chirp during range processing and the imperfect focus of the incoherent jamming pulses during azimuth processing. The increasing similarity of the jamming signal to the radar signal leads to a stronger correlation peak, while the jamming-induced noise level stays reasonably constant within the range of −3.04 dB to −3.05 dB.

#### 3.2.2. Coherent Jammer

**Figure 13:**
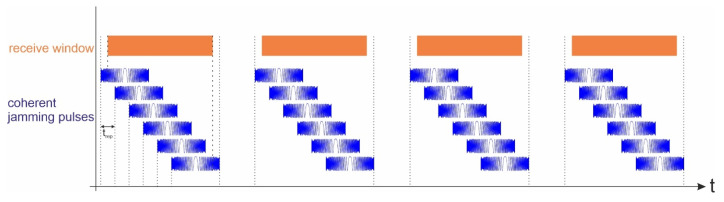
Timing of a coherent jammer that retransmits the incoming radar signal repeatedly during SAR acquisition.

Contrary to the incoherent jammer, a coherent jammer transmits a signal that has identical shape parameters to the original radar signal. The jammer receives the signal coming from the radar sensor and retransmits it repeatedly without any changes in signal shape. Figure 13 shows the corresponding timing scheme during SAR acquisition.

In theory, the coherent jamming signal, which has a shape identical to the radar signal, mainly impacts the SAR processing in the azimuth direction. The reason for this is that the result of range processing for the jamming signal is similar to the result for the radar signal but does not have the correct phase for coherent addition during processing in the azimuth direction.

**Figure 14:**
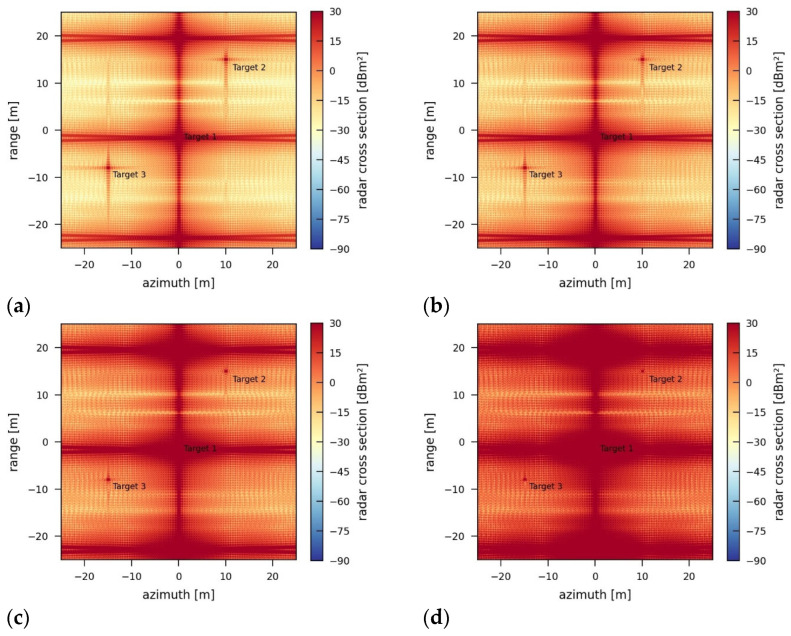
SAR images jammed by a coherent jammer using a pulse repetition frequency of 10 MHz and transmission power of (**a**) 1 W, (**b**) 5 W, (**c**) 50 W, (**d**) 500 W.

Through the simulation of different levels of transmission power during jamming, Figure 14 reveals that the effect of a low-power, but coherent, jamming signal can be significant. This emphasizes the role of the signal phase in jamming strategies. The repetitions of the coherent radar signal used as the jamming signal (see Figure 13) are introduced to the recorded SAR image as strong artifacts (red patterns in Figure 14) resembling defocused point targets. In this way, the signal retransmitted by the jammer is processed like a real radar return from a target that is out of phase. The correlation function during range processing works nominally, and the raw data is correctly focused on the range direction. On the other hand, processing in azimuth is imprecise due to the lack of correct phase information in the jamming signal. This leads to the defocused-like jamming artifacts (false targets) in Figure 14. The defocused false targets caused by the jammer are so pronounced that some of the real point targets in the reference scene are covered by them. Furthermore, depending on the jamming pattern, the target properties, and the two image quality parameters (radar cross section and peak-to-sidelobe ratio (PSLR)) for the targets change locally within the image. By keeping the same radiometric scaling and using the same calibration as for the unjammed reference image, the resulting artifacts are more widely spread over the image with increasing transmission power (Figure 14). These results are tabulated for easy comparison and shown in Table 3.

**Figure 15:**
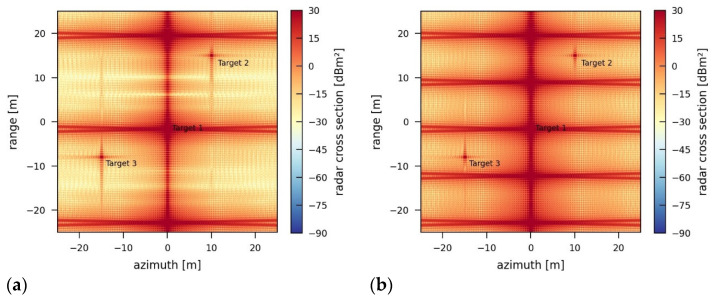

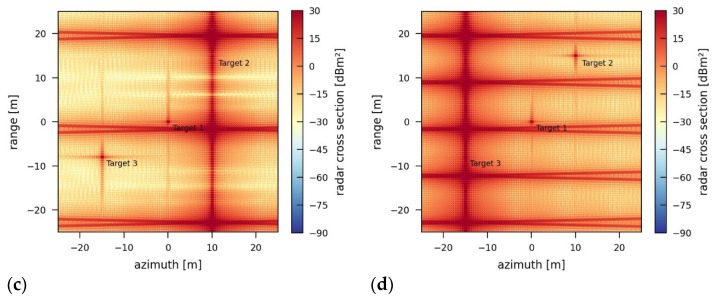
SAR images jammed by a coherent jammer using a transmission power of 1 W; pulse repetition frequency of (**a**) 5 MHz, (**c**) 10 MHz, and (**b**,**d**) 20 MHz; and a jammer position in the azimuth direction of (**a**) 0 m, (**b**) 0 m, (**c**) 10 m and (**d**) −15 m.

When increasing the repetition frequency of the retransmitted pulse, the number of artifacts also rises proportionally to the repetition frequency (Figure 15). A shift of the jammer in the azimuth direction also shifts the resulting artifacts in the image. The exact position and shape of the resulting artifacts depend strongly on the exact phase and timing of the jamming signal, which is not adapted by the jammer in any way. Also, a delay caused by the jammer itself would cause a shift in the position of the artifacts, depending on the jammer’s construction.

#### 3.2.3. Intelligent Jammer

**Figure 16:**
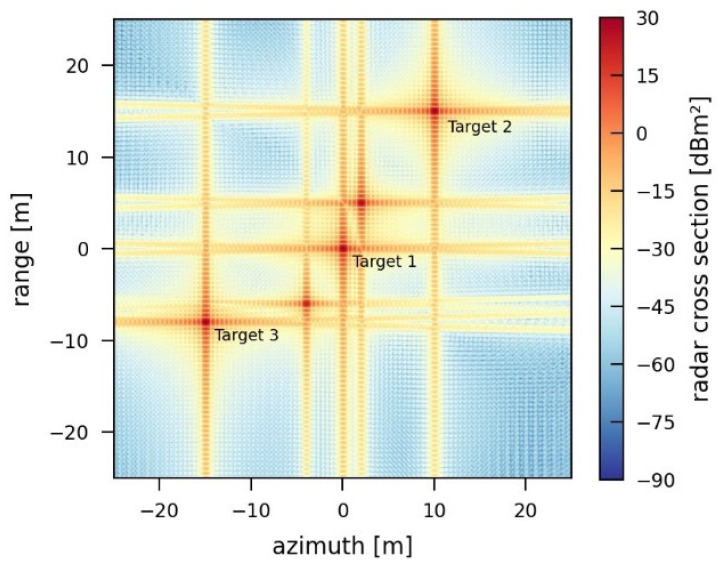
SAR image of the reference scene with an intelligent, constructive jammer for the insertion of two false targets into the image.

The design of an intelligent jammer extends the capabilities of the coherent jammer and includes the adaptation of transmission power, phase, and timing to the original signal before retransmitting the jamming pulse. The jammer receives the radar signal and retransmits a signal of a point target. This type of jammer is a theoretical construct at the moment, as the jamming parameters have to be calculated on the fly. However, advances in technology can make this type of jammer possible in the future. In terms of recording geometry, the corresponding transmission power, timing, and phase are calculated to simulate the behavior of a real target under acquisition. With this method, it is possible to virtually inject false targets or artifacts into the resulting SAR image. Using destructive interference, a backscatter reduction for a real target is also possible. This type of jammer requires sophisticated knowledge of the sensor, its flight path, and the exact acquisition geometry of the scene. The first simulation presented in Figure 16 illustrates the constructive injection of point targets of 500 m^2^ and 150 m^2^. The radar signal is manipulated and retransmitted two times in order to create two false targets with different radar cross sections. The resulting parameters are listed in Table 4.

**Figure 17:**
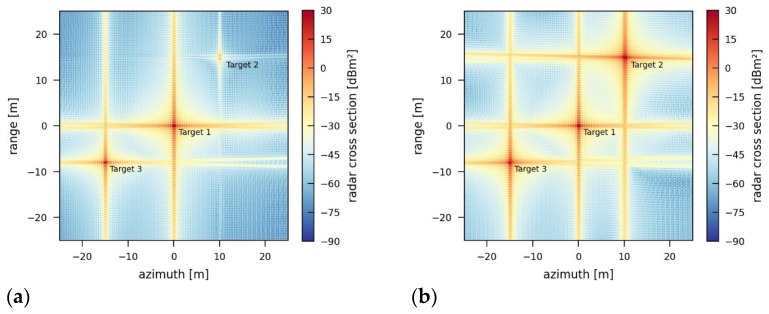
SAR image jammed by an intelligent, destructive jammer for the reduction of two-point targets by 1000 m^2^ (Target 1) and 800 m^2^ (Target 3) (**a**) with exact knowledge of the sensor position and (**b**) with inaccurate knowledge of the sensor position (positioning error: x 6000 m, y, z 100 m).

In contrast, the jammer simulated in Figure 17a additionally rotates the phase of the retransmitted signal by 180° as well as adapts the timing and the phase of the retransmitted signal such that the RCS of the two outer real targets within the scene is reduced. As a result, Target 2 is almost completely removed from the scene, and Target 3 is reduced to a predetermined radar cross section (Table 5a). Figure 17b emphasizes the importance of the exact knowledge of the acquisition geometry in order to remove a point target. If the adapted timing and phase do not exactly match the required values, the reduction or removal of a point target response is not possible (Table 5b). In this case, the assumed positioning error is 6000 m in the x-direction and 100 m in the y- and z-directions. These assumptions are based on the accuracy of available track measurements for satellites (two-line elements) [16].

## 4. Discussion

The proposed categorization of jammers is based on the physical properties of the signal. From the respective simulations, we can understand that the more that is known about the sensor characteristics, the acquisition geometry, and the targets located in the scene, the more precise and effective the jamming becomes. In general, the more similar the jamming signal is to the radar signal, the more effective the jamming. It is important to point out that the simulations are limited to small scenes and a standardized acquisition geometry due to computational constraints. All resulting values are specific to the simulated, generic scene but can be compared to each other. Many parameters can differ in the real world, but the phenomenological results are valid. All simulations assume that the characteristics of the jammer do not change during the image acquisition. In a more real-world scenario, an unintended jamming signal could originate from a communication system. In this case, the characteristics of the jamming signal are not necessarily constant and need further analysis in a potential add-on study.

The simulation of larger scenes would allow the investigation of jamming methods that could have very local effects, such as lines of noise across the image.

The effects caused by jammers can basically be divided into two generic groups. The first group causes a background noise floor over the whole image. For this, the energy is added in an uncorrelated manner to the radar signal. The second group introduces local unwanted effects by affecting the image processing with the jamming signals, whose signal properties are similar to the transmitted SAR signal. In these cases, the correlation of the data during processing in the range direction leads to significant compression of the jamming signal, which would also suggest a categorization of jammers based on the effects in the resulting image as an alternative basis for categorization. Other kinds of categorization can also be suitable, depending on the application. Compared to other studies [17] that define two basic categories, this study deeply examines the physical background of jamming mechanisms for categorization but does not prioritize specific jamming methods [17] or chosen countermeasures [18].

Therefore, a physics-based categorization is chosen because of the solid mathematical foundation needed for traceable simulations and process understanding. This scheme allows the categorization of jammers without the need to know the effects on the resulting image. Hence, it offers an opportunity to understand and create adequate countermeasures for each jamming type depending on the jammer characteristics. In some cases, this kind of categorization might not be unambiguous.

For a jammer, a priori knowledge is helpful to increase the effects of jamming. Knowledge of the signal characteristics employed by the sensor is very important in order to cause significant artifacts in the final SAR images. Detailed information about the acquisition geometry of the sensor is only required for intelligent jammers (third category).

A suggestion for the further development of this topic is to extend the categorization to more subcategories. Of particular interest is the category of jamming by signal shape. This category has the possibility of being divided into subcategories for each physical parameter of the signal, which can be examined under time-dependent assumptions. For example, it would be worth further investigating a jammer that could be categorized somewhere between a coherent and an intelligent jammer. This jammer could retransmit the radar signal but change only the phase of the retransmitted signal. If the timing is correct, it can be hypothesized that the effects of this jammer will be very local, yet distinct.

## 5. Conclusions

This study introduces a categorization of jammers for SAR systems based on a clear physical basis and a solid mathematical description. Jammers, chosen according to the categorization, were simulated, and their effects on the resulting SAR images are described and shown in Section 3. The three main categories are as follows: jamming by transmission power, jamming by signal shape, and intelligent jamming.

The resulting effects on the image differ based on the jammer type. This leads to the conclusion that the effects in the image cannot be uniquely mapped to a type of jammer because multiple types of jamming can cause similar effects in the image. Hence, an effect-based categorization would not be unique in mapping. Jamming by transmission power usually causes a rise in the noise floor, which is spread homogeneously over the image. The main characteristic of this type of jamming is that the noise level increases with the additional energy from the jamming signal during image acquisition. Jamming by signal shape has an effect on the SAR processing and depends on the similarity of the jamming signal to that of the radar signal. The effect on the processed image ranges from a heightened noise floor, seen as background noise, to the generation of unfocused responses, which mimic point target scattering. Moreover, an intelligent jammer is able to generate false targets virtually as well as reduce the signal intensity of real targets by retransmitting signals fully adapted to the image acquisition geometry. These results are the outcome of simulations based on a generic scene. As a next step, the intention is to test the categorization on scenarios closer to the real world, followed by the use of real sensor data.

## Figures and Tables

**Table 1:** Basic parameters of the synthetic reference scenario.

Scene Size (Az, Rg)	50 m × 50 m
Sensor altitude	500 km
Incidence angle	45 deg
RCS Point Targets	30 dbm^2^
Radar signal transmission power	2000 W
Carrier frequency	9.6 GHz
Signal bandwidth	424 MHz
Ground resolution (Az, Rg)	0.5 m × 0.5 m
Pulse duration	5 µs
Pulse repetition frequency	17.29 kHz

**Table 2:** Target properties and image quality parameters of the reference scenario for simulations.

	Target 1	Target 2	Target 3
Position	0.00 m, 0.00 m	10.00 m, 15.00 m	−15.00 m, −8.00 m
Radar cross section	1000.00 m^2^	999.68 m^2^	999.93 m^2^
Resolution (Az, Rg)	0.48 m × 0.45 m	0.48 m × 0.45 m	0.48 m × 0.45 m
PSLR (Az, Rg)	16.69 dB, 13.27 dB	16.69 dB, 13.27 dB	16.69 dB, 13.27 dB
ISLR (Az, Rg)	13.85 dB, 10.12 dB	13.85 dB, 10.11 dB	13.85 dB, 10.12 dB

**Table 3:** Target properties and image quality parameters for coherent jammers of (**a**) 1 W, (**b**) 5 W, (**c**) 50 W, and (**d**) 500 W added to the reference scene.

		Target 1	Target 2	Target 3
(**a**)	Position	0.00 m, 0.00 m	10.00 m, 15.00 m	−15.00 m, −8.00 m
	Radar cross section	not detectable	1029.94 m^2^	995.11 m^2^
	Resolution (Az, Rg)	not detectable	0.47 m × 0.44 m	0.48 m × 0.44 m
	PSLR (Az, Rg)	not detectable	16.64 dB, 3.30 dB	−2.06 dB, 5.67 dB
	ISLR (Az, Rg)	not detectable	13.72 dB, 3.32 dB	13.87 dB, 9.58 dB
(**b**)	Position	0.00 m, 0.00 m	10.00 m, 15.00 m	−15.00 m, −8.00 m
	Radar cross section	not detectable	1067.98 m^2^	989.18 m^2^
	Resolution (Az, Rg)	not detectable	0.46 m × 0.43 m	0.48 m × 0.45 m
	PSLR (Az, Rg)	not detectable	11.98 dB, −3.3 dB	−9.10 dB, −1.36 dB
	ISLR (Az, Rg)	not detectable	12.99 dB, −2.16 dB	13.78 dB, 8.45 dB
(**c**)	Position	not detectable	not detectable	−15.00 m, −7.95 m
	Radar cross section	not detectable	not detectable	978.33 m^2^
	Resolution (Az, Rg)	not detectable	not detectable	0.48 m × 0.45 m
	PSLR (Az, Rg)	not detectable	not detectable	−19.68 dB, −11.42 dB
	ISLR (Az, Rg)	not detectable	not detectable	12.69 dB, 4.91 dB
(**d**)	Position	not detectable	not detectable	−15.00 m, −7.85 m
	Radar cross section	not detectable	not detectable	1085.24 m^2^
	Resolution (Az, Rg)	not detectable	not detectable	0.43 m × 0.43 m
	PSLR (Az, Rg)	not detectable	not detectable	−27.67 dB, −20.97 dB
	ISLR (Az, Rg)	not detectable	not detectable	4.75 dB, −0.49 dB

**Table 4:** Target properties and image quality parameters for an intelligent jammer creating two virtual false targets within the scenario.

	Target 1	False Target 1	False Target 2
Position	0.00 m, 0.00 m	2.00 m, 5.00 m	−4.00 m, −6.00 m
Radar cross section	1000.12 m^2^	499.97 m^2^	149.59 m^2^
Resolution (Az, Rg)	0.48 m × 0.44 m	0.48 m × 0.44 m	0.48 m × 0.44 m
PSLR (Az, Rg)	16.69 dB, 13.26 dB	16.68 m^2^, 13.28 dB	16.66 dB, 13.29 dB
ISLR (Az, Rg)	13.86 dB, 10.16 dB	13.85 dB, 10.18 dB	13.81 dB, 10.19 dB

**Table 5:** Target properties and image quality parameters for an intelligent, destructive jammer for the reduction of two-point targets by 1000 m^2^ (Target 1) and 800 m^2^ (Target 3) (**a**) with exact knowledge of the sensor position and (**b**) with inaccurate knowledge of the sensor position.

		Target 1	Target 2	Target 3
(**a**)	Position	0 m, 0 m	10.00 m, 15.00 m	−15.00 m, −8.00 m
	Radar cross section	999.80 m^2^	not detectable	200.13 m
	Resolution (Az, Rg)	0.48 m × 0.44 m	not detectable	0.48 m × 0.44 m
	PSLR (Az, Rg)	16.69 dB, 13.26 dB	not detectable	16.69 dB, 13.25 dB
	ISLR (Az, Rg)	13.85 dB, 10.16 dB	not detectable	13.85 dB, 10.14 dB
(**b**)	Position	0 m, 0 m	10.25 m, 14.85 m	−15.05 m, −8.10 m
	Radar cross section	999.96 m^2^	597.26 m^2^	429.67 m^2^
	Resolution (Az, Rg)	0.48 m × 0.44 m	0.75 m × 0.48 m	0.47 m × 0.41 m
	PSLR (Az, Rg)	16.69 dB, 13.26 dB	14.31 dB, 12.93 dB	15.47 dB, 8.86 dB
	ISLR (Az, Rg)	13.85 dB, 10.16 dB	12.56 dB, 8.64 dB	12.84 dB, 6.55 dB

## Data Availability

The original data presented in this study are included in the article. Further inquiries can be directed to the corresponding author.

## Abbreviations

The following abbreviations are used in this manuscript:

Az	Azimuth Direction
ISLR	Integrated Sidelobe Ratio
JINL	Jamming-Induced Noise Level
PSLR	Peak Sidelobe Ratio
RCS	Radar Cross Section
Rg	Range Direction
SAR	Synthetic Aperture Radar
